# Prenatal Intervention with Partial Meal Replacement Improves Micronutrient Intake of Pregnant Women with Obesity

**DOI:** 10.3390/nu11051071

**Published:** 2019-05-14

**Authors:** Suzanne Phelan, Barbara Abrams, Rena R. Wing

**Affiliations:** 1 Department of Kinesiology & Public Health, California Polytechnic State University, 1 Grand Ave, San Luis Obispo, CA 93407, USA; 2 Division of Epidemiology, University of California at Berkeley School of Public Health, 2121 Berkeley Way #5302, Berkeley, CA 94720-7360, USA; babrams@berkeley.edu; 3 Warren Alpert Medical School at Brown University Department of Psychiatry and Human Behavior, 197 Richmond Street, Providence, RI 02906, USA; rwing@lifespan.org

**Keywords:** prenatal intervention, meal replacements, randomized clinical trial, lifestyle intervention, obesity, RDA, micronutrients

## Abstract

A behavioral lifestyle intervention with partial meal replacement reduced excess gestational weight gain in ethnically diverse women with overweight/obesity, but the effects on micronutrient intake remained unknown. A secondary analysis of a randomized, controlled trial tested whether the intervention improved micronutrient intake relative to usual care. Pregnant women (*n* = 211; 30.5 years of age, body mass index, BMI, of 32.0 kg/m^2^) were enrolled and randomized within site and ethnicity (40% were Hispanic) into intervention (*n* = 102) or usual care (*n* = 109) groups. Two 24 h dietary recalls were conducted on random days at study entry and late pregnancy (35–36 weeks gestation). Nutrient adequacy was defined using the Estimated Average Requirement cut-point method. At study entry and including prenatal vitamins, ≥90% of participants reported inadequate intake of vitamins D and E and iron; 40–50% reported inadequate intake of calcium, protein, vitamins A, C, B_6_, folate, magnesium, and zinc. From study entry to late pregnancy, the behavioral intervention with partial meal replacement increased the overall intake of vitamins A, E, and D and copper and reduced the odds of inadequate intake of calcium (odds ratio (OR) = 0.37 (0.18, 0.76)), vitamins A (OR = 0.39 (0.21, 0.72)) and E (OR = 0.17 (0.06, 0.48)), and magnesium (OR = 0.36 (0.20, 0.65)). A behavioral intervention with partial meal replacement during pregnancy improved the intake of several micronutrients in Hispanic and non-Hispanic women with overweight/obesity.

## 1. Introduction

Maternal pre-pregnancy obesity and excess gestational weight gain are well established risk factors for several adverse short- and long-term maternal and child health outcomes, including pregnancy complications, diabetes, obesity, and cardio-metabolic comorbidities [1]. Compounding risks, pregnant women with obesity are more likely than those with normal weight to have micronutrient insufficiency [2,3,4], conferring additional potential risks of pregnancy complications and chronic conditions in later life [5,6]. 

The Academy of Nutrition and Dietetics recommends that the optimal prenatal diet should limit overconsumption yet prevent micronutrient insufficiency [5]. A varied and balanced diet, rich in fruits, vegetables, and whole grains is recommended. Several studies have shown that comprehensive lifestyle interventions that target reduced calorie intake and balanced nutrition can effectively reduce excess gestational weight gain in women with obesity [7], but less is known about intervention effects on micronutrient intake. Micronutrient needs increase during pregnancy, particularly for folic acid, iron, zinc, calcium, vitamin C, and vitamin D [8]. While supplementation can reduce micronutrient deficiencies and some associated maternal and fetal complications [9,10,11,12], adherence to prenatal multivitamins may be limited by gastrointestinal distress or nausea [13]. The provision of micronutrient-rich foods [14,15] and nutrition educational interventions [16] can also improve dietary quality and pregnancy outcomes, but few randomized clinical trials have been done, particularly in pregnant women with obesity who are at risk of micronutrient insufficiency [15].

Healthy Beginnings/Cominezos Saludables was a randomized clinical trial designed to test the efficacy of a partial meal replacement program versus usual care to reduce excessive gestational weight gain in 257 pregnant women with overweight or obesity. The intervention significantly reduced excess gestational weight gain, which was related to increased use of meal replacements during pregnancy [17]. No significant treatment effect was seen on energy or macronutrient composition [17]. Since the provided meal replacements were fortified with vitamins and minerals, it is possible that they could address underlying micronutrient inadequacies in pregnant women with overweight or obesity. The purpose of this study was to determine if the behavioral intervention with partial meal replacement compared with usual care improved micronutrient intake of pregnant women with overweight and obesity.

## 2. Materials and Methods

### 2.1. Design

Healthy Beginnings/Comienzo Saludables was a randomized controlled trial conducted at California Polytechnic State University, San Luis Obispo, California, and at the Miriam Hospital with Women & Infants Hospital in Providence, Rhode Island, and was part of the Lifestyle Interventions for Expectant Moms (LIFE-Moms) consortium [18]. Clinical Trial Registry Number: ClinicalTrials.gov, www.clinicaltrials.gov, NCT01545934.

### 2.2. Participants

**Figure 1:**
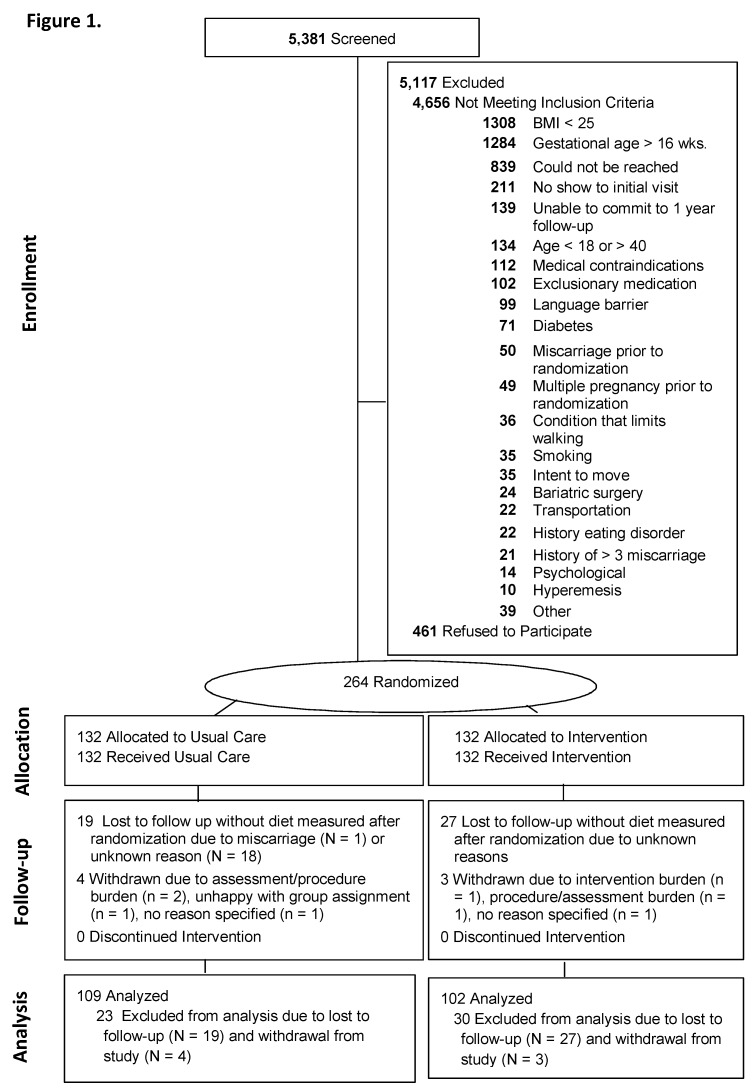
Participant flow and Retention in Healthy Beginnings/Comienzos Saludables. BMI = body mass index.

The study was conducted in accordance with the ethical principles of research; the protocol was approved by the Institutional Review Boards of California Polytechnic State University (2018-264) and the Miriam Hospital (2144-11), and all participants provided written informed consent. As previously described [17,19], recruitment occurred November 2012 and October 2015 in California and Rhode Island. Eligibility criteria included gestational age between 9 and 16 weeks, body mass index (BMI) ≥25, being English or Spanish-speaking, age ≥18 years, and singleton pregnancy. Participants were excluded if they had glycosylated hemoglobin (Hb A1c) ≥ 6.5 or self-reported major health diseases (e.g., heart disease, cancer, renal disease, and diabetes), and other (Figure 1). Of the 5381 screened women, 24% were excluded due to BMI < 25, and 24% were excluded due to gestational age >16 weeks. Other prevalent reasons for exclusions are shown in Figure 1. 

### 2.3. Interventions

In this two-site trial, randomization was computer-generated by the study statistician, and women were randomly assigned within site and ethnicity (Hispanic versus non-Hispanic) to one of the two treatment conditions: (1) usual care or (2) behavioral lifestyle intervention with partial meal replacement. 

### 2.4. Usual Care

Participants in the usual care group received all aspects of usual care offered by their prenatal care providers [20]. Usual prenatal care visits typically occur monthly until 28 weeks of gestation, bi-weekly between 28 and 36 weeks of gestation, and weekly until delivery. Also, in this group at the time of study randomization, participants attended a ~20 min welcome visit with a study interventionist, providing general information about healthy eating, physical activity, and the Institte of Medicine (IOM)recommendations for total gestational weight gain [21]. Study interventionists were bilingual registered dietitians or counselors with degrees in nutrition, community health, psychology, kinesiology, or a related field. Participants received study newsletters with general information about pregnancy-related health, including consuming prenatal vitamins, quitting smoking, planning to breastfeed, and fetal growth. 

### 2.5. Behavioral Lifestyle Intervention with Partial Meal Replacement during Pregnancy

Participants in the intervention group received all aspects of usual care plus a behavioral lifestyle intervention designed to prevent excessive weight gain during pregnancy. As described previously [17], the intervention targeted healthy eating, activity, and behavioral strategies. Each woman received ~20 min, individual, face-to-face counseling sessions with a study interventionist every two weeks until 20 weeks of gestation and then monthly visits until delivery. Women were encouraged to gain approximately one-half pound (0.23 kg) per week, on the basis of the 2009 IOM guidelines [21]. To promote adherence to weight gain guidelines, women were provided with a structured meal plan [22] that was individually tailored to meet each participant’s self-reported dietary needs, including food aversions, cravings, lactose intolerance, and specialized diets, such as vegetarianism. The plan provided a caloric prescription of ~18 kcal/kg of body weight at study entry [23] and consisted of 30% of calories from fat, 15–20% from protein, and 50–55% from carbohydrates [24]. Women were instructed to replace two meals with a provided meal replacement shake or bar and to consume at least one meal of regular foods and two to four healthy snacks each day. The meal replacement products were provided free of charge at every intervention visit and in the quantities needed until the next scheduled intervention visit. The study’s meal replacement options were selected at study onset by the investigators after an analysis of various meal plan scenarios that considered the micronutrient and macronutrient composition of specific meal replacement products, the participant’s use of prenatal vitamins, the intervention’s calorie and nutritional goals, and the current micronutrient and macronutrient recommendations for pregnant women [24]. Options included organic and lactose-free drinks and bars in a variety of flavors and brands (Supplemental Table S1). 

### 2.6. Outcome Assessments

Assessments were conducted early in pregnancy (between 9 and 16 weeks) and at 35–36 weeks of gestation. The participants received $25 for completing each assessment. The assessment staff was masked to randomization to minimize potential bias. Dietary intake was assessed at study entry and 35–36 weeks of gestation using interview-administered 24 h recalls on two random days over a week and completed using the National Cancer Institute Automated Self-Administered 24 h recall (ASA-24; http://riskfactor.cancer.gov/tools/instruments/asa24.html) [25]. The ASA-24 provided values (combined from food, beverages, and supplements) for thiamin, riboflavin, niacin, vitamin B6, folate, vitamin B12, vitamin C, vitamin A, vitamin E, iron, zinc, calcium, magnesium, phosphorous, copper, selenium, water, energy, carbohydrate, total fat, and protein and included intake of supplements [26]. Dietary intake was categorized as meeting or not meeting the Recommended Daily Allowance (RDA) based on the Estimated Average Requirement (EAR) of the Institute of Medicine (IOM) for pregnant women [27,28,29,30,31,32]. The RDA represents an estimate of the average daily intake level sufficient to meet the nutrient requirements for 97–98% of healthy individuals. To assess the prevalence of inadequacy, the cut-point method was used [29,33] to classify individuals with intakes below versus at or above the median EAR considered needed for half of the individuals in the population (Table 1) [27,28,29,30,31,32,34,35]. For micronutrients in which an RDA and EAR had not been established (i.e., vitamin K, choline, potassium, sodium), cutoffs based on Adequate Intakes (AI) were used, albeit interpreted with less confidence [27,28,29,30,31,32]. The ASA-24 was also used to measure meal replacement intake, quantified as the total number of meal replacement products including shakes and bars that were consumed each day, on average, during the assessment period. 

Weight and height were assessed in duplicate to the nearest 0.1 kg or 0.1 cm using a calibrated standard digital scale and stadiometer with the participant in lightweight clothing without shoes. Heritage and ethnicity were assessed by self-report using questionnaires with fixed categories. Marital status, income, education, employment status, and childbearing history were also assessed by self-report questionnaires. Gestational age in weeks at study entry was measured via clinical ultrasound. 

### 2.7. Statistical Methods

#### Analysis Plan

To compare participants in the two groups and completers versus non-completers, Independent *t*-test for continuous variables and Pearson χ2 test or exact tests for categorical variables were used. To test if the intervention versus usual care affected micronutrient values, a repeated measures analysis of variance was used. The models included the terms treatment group and group × time interactions (fixed effect) and a priori defined covariates that included weeks of gestation at randomization, age, ethnicity (Hispanic versus non-Hispanic), parity (multiparity versus primiparity), study entry BMI category (overweight versus obese), household family income (≥50,000/year versus <50,000/year), and baseline value of variable of interest [36]; site (California versus Rhode Island) was also included as a fixed effect. (A sensitivity analysis that divided the income into four categories and also included education did not alter the results). To test whether the intervention versus usual care improved micronutrient adequacy (meeting versus not meeting RDA’s EAR cutpoint), logistic regression was used that included the treatment group and the same covariates. Within the intervention group alone, number of meal replacement products/day and changes in micronutrients status were also analyzed via Pearson’s partial correlations, adjusting for the same covariates. Statistical significance was set to *p* < 0.05. The SPSS (23.0.0; IBM Corporation; Armonk, NY, USA) statistical package was used for all analyses. 

## 3. Results

Figure 1 summarizes the participant flow and retention into Healthy Beginnings/Comienzos Saludables. Participant characteristics were well balanced by randomized group (Table 2). At the 35–36 weeks of gestation visit, 82.1% (211/257) of participants completed the dietary assessment, including 85.2% (109/128) of usual care and 79.1% (102/129) of intervention participants, with no statistically significant (*p* = 0.55) differences in retention by group. The demographic characteristics (site, group, BMI, age, education, parity, weeks of gestation at randomization) did not significantly differ between participants who completed and those who did not complete the 35–36 weeks gestation visit. Completers were more likely than non-completers to have adequate intake of phosphorus (38/43 versus 209/211; chi square test *p* = 0.002), niacin (32/46 versus 187/211; *p* = 0.002), riboflavin (33/46 versus 185/211; *p* = 0.011), and thiamin (23/46 versus 144/211; *p* = 0.03); no other significant differences were observed.

### 3.1. Intervention Effects on Micronutrient Intake

When examining the average changes in micronutrients from study entry to 35–36 weeks gestation (Table 3), significant group × time interactions indicated that the intervention relative to usual care increased the average intake of vitamins A (178.3 versus 34.6, μg/day, respectively; *p* = 0.0001), E (1.9 versus −0.3 mg/day; *p* = 0.0001), and K (23.4 versus −11.7 μg/day; *p* = 0.04). In addition, the intervention significantly increased (relative to usual care) the intakes of vitamin D (1.5 versus 0.5 μg/day; *p* = 0.045) and copper (259.2 versus −34.5, μg/day; *p* = 0.001) and significantly decreased the intake of selenium (−11.0 versus −7.2 μg/day; *p* = 0.002). 

### 3.2. Intervention Effects on Micronutrient Adequacy Based on the RDAs

Study Entry. Despite the prevalent intake of prenatal vitamins (97%, Table 2), significant proportions of participants reported inadequate intakes of nearly every micronutrient on the basis of the EARs for pregnant women (Table 4). The vast majority (≥90%) reported inadequate intakes of fiber, vitamin D, Vitamin E, iron. Nearly half (between 40 and 50%) of the participants also reported inadequate intakes of calcium, protein, vitamin A, vitamin C, vitamin B_6_, folate, magnesium, and zinc. On the basis of AI cutoffs, inadequate intakes of choline and vitamin K were also quite prevalent (Table 4). Few participants reported intakes that were at or above the recommended tolerable limit for the micronutrients. Exceptions were that a majority of participants had higher than recommended as tolerable levels of sodium (170/211; 81.0%), and a minority had excess magnesium (39/211; 18.5%), folate (12/211; 5.7%), and niacin (19/211; 9.0%) at study entry (Table 5).

Study Entry to 35–36 Weeks of Gestation. Significant group x time interactions were observed that indicated that the intervention significantly reduced the odds at 35–36 weeks of gestation of inadequate intake based on the EARs for calcium (odds ratio (OR) = 0.34 (0.18, 0.76) *p* = 0.007), vitamin A (OR = 0.39 (0.21, 0.72) *p* = 0.003;), vitamin E (OR = 0.17 (0.06, 0.48) *p* = 0.001), and magnesium (OR = 0.36 (0.20, 0.65) *p* = 0.001), as shown in Table 4. Based on AIs, the intervention also decreased the odds of inadequate intake of vitamin K (OR = 0.49 (0.26, 0.91)) and increased the odds of inadequate intake of choline (OR = 5.0 (1.0, 24.6) *p* = 0.04).

From study entry to 35–36 weeks of gestation, the intervention reduced the odds of intake above tolerable limits for sodium (OR = 0.47 (0.24, 0.91) *p* = 0.026) and increased the odds of intake above tolerable limits for magnesium (OR = 2.0 (1.0, 3.7); *p* = 0.038) (Table 5).

### 3.3. Intervention Adherence

**Figure 2:**
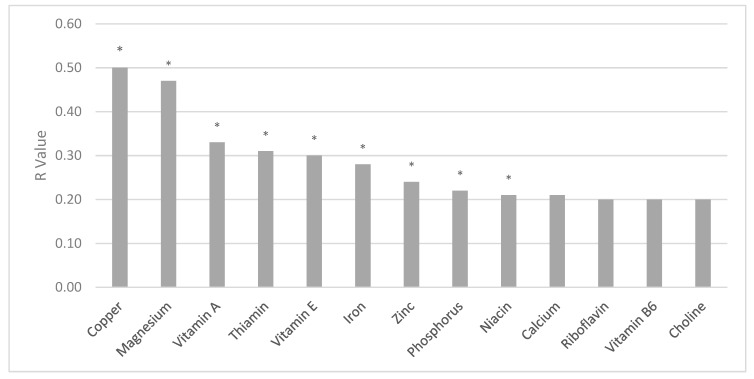
R values for changes in daily meal replacement servings/day and micronutrients from study entry to 35 weeks gestation among the intervention participants. * *p* < 0.05.

From baseline to weeks 35–36, the intervention increased the average number of meal replacement products reported each day by an additional 0.63 (SD 0.83) products/day. Increased reported intake of meal replacement products/day was significantly related to increased intake of micronutrients (Figure 2), including vitamin A (*r* = 0.33; *p* = 0.002), vitamin E (*r* = 0.30; *p* = 0.004), niacin (*r* = 0.21; *p* = 0.043), thiamin (*r* = 0.31; *p* = 0.003), copper (*r* = 0.50; *p* = 0.0001), iron (*r* = 0.28; *p* = 0.008), magnesium (*r* = 0.47; *p* = 0.0001), phosphorus (*r* = 0.22; *p* = 0.04), and zinc (*r* = 0.24l *p* = 0.02). Trend positive partial correlations were observed for calcium (*r* = 0.21; *p* = 0.05), riboflavin (*r* = 0.20; *p* = 0.058), vitamin B_6_ (*r* = 0.20; *p* = 0.06), and choline (*r* = 0.20; *p* = 0.06), and a trend inverse correlation was observed for vitamin K (*r* = −0.18; *p* = 0.08) (Figure 2). No significant correlations were observed for changes in meal replacements and vitamin C, K, folic acid, B_12_, selenium, potassium, or sodium.

## 4. Discussion

A prenatal meal replacement intervention that reduced excess gestational weight gain also improved the micronutrient intake of pregnant women with overweight and obesity. At study entry, despite the intake of prenatal vitamins, more than 90% of participants reported inadequate intakes of vitamin E, and about 50% reported inadequate intakes of calcium, vitamins A and K, and magnesium. By 35 weeks of gestation, the prenatal lifestyle intervention with partial meal replacement had cut by more than half the odds of these micronutrient insufficiencies. Given the association between inadequate levels of nutrients during pregnancy and later adverse maternal and child health conditions [5], these findings highlight an additional potential benefit of the lifestyle intervention that reduced excess gestational weight gain [17].

At study entry, the vast majority of participants (81%) had higher than recommended intakes for sodium. By 35 weeks gestation, the intervention reduced by nearly 90% the odds of excess sodium intake based on AI cutoffs. The effects of excess sodium intake during pregnancy remain unclear [37]. Animal and some human studies have suggested that excess sodium may negatively affect the immune system [38] and placental functioning [37,39,40] and contribute to the development of high blood pressure and pre-eclampsia [41]. However, mixed findings and lack of evidence that restricting sodium during pregnancy has any long-term health benefits to mothers or children have led the current guidelines not to recommend sodium restriction during pregnancy, unless a woman has high blood pressure [42,43]. In the current trial, the intervention did not have a significant effect on lowering blood pressure [17], despite the reductions in sodium intake reported here.

The intervention-related increases in micronutrient intake generally occurred without promoting an excess in micronutrients—with the exception of magnesium. The intervention increased the odds of exceeding the recommended tolerable limit for intake of magnesium (i.e., 350 mg/day). The upper limits are not intended to be a recommended “cap” but rather a level of intake that most individuals could likely tolerate [29,30,34,35]. Nevertheless, high amounts of magnesium could result in diarrhea, nausea, and abdominal cramping [29,30,34,35], which were not measured in the current study.

Also of note, the intervention increased the odds of inadequate intake of choline. Choline does not yet have an established EAR, so cutoffs were based on AI, which should be interpreted with less confidence [27,28,29,30,31,32]. Nevertheless, the reasons for an intervention-related decline in choline remain unclear. The meal replacements likely did not contain adequate amounts of choline and might have displaced typical consumption of choline-containing foods, such as eggs and milk. However, group differences in milk intake were not observed (data not shown). Adequate intake of dietary choline may be important for optimal fetal outcome (birth defects, brain development) and for maternal liver and placental function [44]. Future research of the intervention approach should consider the promotion of both egg and milk consumption or other choline-rich foods or a diet supplement that might assist women in the intervention in meeting the daily choline intake recommendations.

This study is the first to examine whether a prenatal lifestyle intervention with partial meal replacement that reduced excess gestational weight gain had positive effects on micronutrient intake of pregnant women with overweight and obesity. The study’s strengths include its randomized design, diverse population, blinded assessors, and two repeated, random, and interview-administered 24 h recalls to measure the dietary intake. Limitations of this study include the fact that the assessment of micronutrients was solely based on self-report, which could have overestimated the proportions with inadequate intake [45]. Also, data were lacking for some micronutrients, including molybdenum and iodine. Also, for micronutrients in which an RDA and EAR had not been established (i.e., vitamin K, choline, potassium, sodium), cutoffs were based on AIs, which should be interpreted with less confidence, since the amount and quality of data currently available may not be sufficient to make reliable estimates [46]. The study design tested a treatment “package” and did not allow for isolation of the independent contribution of meal replacements from other intervention components. Future randomized clinical trials are needed to tease apart the intervention “package” and identify the independent contribution of meal replacements and other intervention components on improving micronutrient adequacy. Only 82% of the original sample completed the dietary assessment, although the characteristics of completers versus non-completers were similar. Also, the study’s sample size was not powered to examine the effect of changes in micronutrients on maternal and fetal complications.

## 5. Conclusions

A comprehensive prenatal lifestyle intervention that reduced excess gestational weight gain [17] also improved micronutrient intake and reduced the odds of inadequate intake of several micronutrients. A low nutritional quality of the diet and inadequate intakes of micronutrients can have significant consequences for both the mother and the developing fetus. Future research is now needed to examine the generalizability and effectiveness of this prenatal lifestyle modification program in other populations and settings.

## Figures and Tables

**Table 1:** Estimated average requirements (EAR) ^1^ for pregnancy.

Nutrient	Recommended Amount
Total Water (L/d) **	3.0 **
Calcium (mg/d)	800
CHO (g/d)	135
Fiber (g/d) **	28 **
Added sugars	≤25% of TE
Protein (g/kg/d)	0.88
Vit A μg, RAE/d	550
Vit C (mg/d)	70
Vit D (μg/d)	10
Vit E (mg/d)	12
Vit K μg/d **	90 **
Thiamin (mg/d)	1.2
Riboflavin (mg/d)	1.2
Niacin (mg/d)	14
Vit B6 (mg/d)	1.6
Folate (μg/d)	520
Vit B12 (μg/d)	2.2
Choline mg/d **	450 **
Copper (μg/d)	800
Iron (mg/d)	22
Magnesium (mg/d)	290
Phosphorus (mg/d)	580
Selenium (μg/d)	49
Zinc (mg/d)	9.5
Potassium mg/d **	4.7 **
Sodium g/d **	1.5 **

Abbreviations: Vit: vitamin; TE: total energy; d: day; RAE: retinol activity equivalents; CHO: carbohydrates: ^1^ EAR is the average daily nutrient intake level estimated to meet the requirements of half of the healthy individuals who are pregnant and aged 19–30 years [27,28,29,30,31,32]. Vitamin A based on RAEs; vitamin E based on α-tocopherol; niacin expressed as niacin equivalents; folate expressed as folate equivalents. Food and Nutrition Board, Institute of Medicine, National Academies reports may be accessed via www.nap.edu [27,28,29,30,31,32], ** Adequate Intakes (AI) because EARs have not been established; this cutoff is made with less confidence [27,28,29,30,31,32].

**Table 2:** Baseline characteristics of the participants by condition.

Characteristic	Total *n* = 211	Usual Care *n* = 109	Intervention *n* = 102
Age, years, Mean (SD)	30.5 (5.3)	30.0 (5.6)	31.0 (4.9)
Hispanic/Latino, No. (%)			
Yes	85 (40.3)	43 (39.4)	42 (41.2)
No	126 (59.7)	66 (60.6)	60 (58.8)
Heritage, No. (%) (participants could select multiple)			
American Indian or Alaskan Native	7 (3.3)	3 (2.8)	4 (3.9)
Asian	2 (0.9)	0 (0)	2 (2.0)
Black or African American	15 (7.1)	7 (6.4)	8 (7.8)
Native Hawaiian or Pacific Islander	4 (1.9)	3 (2.8)	1 (1.0)
White	131 (62.1)	67 (61.5)	64 (62.7)
Other	60 (28.4)	30 (27.5)	30 (29.4)
Marital Status, No. (%)			
Married or living with significant other	148 (70.1)	97 (89.0)	51 (50.0)
Never married/divorced/widowed	63 (29.9)	12 (11.0)	51 (50.0)
Annual household Income $, No. (%)			
<$24,999	49 (23.2)	28 (25.7)	21 (20.6)
$25,000–49,999	62 (29.4)	30 (27.5)	32 (31.4)
$50,000–99,999	60 (28.4)	30 (27.5)	30 (29.4)
≥$100,000	40 (19.0)	21 (19.3)	19 (18.6)
Education, No. (%)			
High school or less	48 (22.7)	29 (26.6)	19 (18.6)
Some college/College	130 (61.6)	62 (56.9)	68 (66.7)
Post-graduate work	33 (15.6)	18 (16.5)	15 (14.7)
Employment, No. (%)			
Employed Full Time (at least 35 hours/week)	121 (57.3)	62 (56.9)	59 (57.8)
Employed Part-Time (less than 35 hours/week)	38 (18.0)	22 (20.2)	16 (15.7)
Unemployed	52 (24.6)	25 (22.9)	27 (26.5)
Childbearing history, No. (%)			
Primiparous	53 (25.1)	25 (22.9)	28 (27.5)
Multiparous	154 (73.0)	82 (75.2)	72 (70.6)
Weeks of gestation at study entry, Mean (SD)	13.6 (1.7)	13.5 (1.9)	13.8 (1.5)
Weight, kg, at study entry, Mean (SD)	84.9 (16.5)	86.1 (17.9)	83.6 (14.8)
BMI, kg/m^2^, at study entry, Mean (SD)	32. (5.3)	32.5 (5.4)	32.1 (5.3)
Weight status			
Overweight, No. (%)	86 (40.8)	42 (38.5)	44 (43.1)
Obese, No. (%)	125 (59.2)	67 (61.5)	58 (56.9)
Preconception weight, Mean (SD)	83.0 (16.5)	81.9 (14.8)	84.1 (17.9)
Preconception weight status			
Overweight, No. (%)	94 (44.5)	44 (40.4)	50 (49.0)
Obese, No. (%)	114 (54.0)	63 (57.8)	51 (50.0)
Weight gain from preconception to study entry, kg, Mean (SD)	1.9 (4.4)	1.8 (3.3)	19 (5.0)
Daily prenatal vitamin intake, No. (%)	105 (96.7%)	105 (96.3)	99 (97.0)

Abbreviations: SD: standard deviation; BMI is calculated as weight in kilograms divided by the square of height in meters. Y: years. No.: number.

**Table 3:** Micronutrient intake from early pregnancy (baseline) to 35 weeks of gestation by treatment group.

	EAR	Usual Care; *n* = 109 Mean (SD)		Intervention; *n* = 102 Mean (SD)		Statistical Results ^1^	
		Baseline	35 Weeks	Baseline	35 Weeks	T	G × T
Total Water, L/d	3.0 **	2.6 (0.9)	2.8 (1.1)	2.5 (0.8)	2.8 (0.9)	0.20	0.72
Calcium, mg/d	800	973.5 (355.8)	1026.0 (387.8)	928.9 (352.1)	1097.2 (378.0)	0.06	0.14
CHO, g/d	135	219.6 (68.3)	225.1 (71.9)	219.7 (75.6)	217.7 (63.3)	0.001	0.783
Fiber, g/d	28 **	16.5 (6.3)	15.6 (6.6)	16.7 (6.9)	14.2 (6.4)	0.02	0.07
Added sugars, % TE	<25% of TE	9.6 (4.6)	10.3 (7.0)	10.5 (6.1)	12.3 (10.3)	0.28	0.35
Protein, g/kg/d,	0.88	0.9 (0.3)	0.8 (0.3)	0.9 (.31665)	0.8 (0.3)	0.92	0.76
**Vit A μg, RAE/d·**	**550**	**631.2 (336.6)**	**667.6 (372.3)**	**720.0 (378.9)**	**898.0 (402.9)**	**0.10**	**0.0001**
Vit C, mg/d	70	90.1 (62.4)	78.0 (57.3)	103.3 (70.5)	94.6 (64.8)	0.03	0.09
**Vit D, μg/d**	**10**	**4.1 (2.6)**	**4.6 (2.9)**	**4.0 (2.5)**	**5.5 (3.3)**	**0.55**	**0.045**
**Vit E, mg/d**	**12**	**7.2 (4.4)**	**6.9 (3.1)**	**7.3 (3.7)**	**9.2 (5.0)**	**0.03**	**0.0001**
**Vit K, μg/d**	90 *	**111.8 (108.6)**	**100.0 (105.0)**	**116.1 (114.3)**	**140.0 (175.3)**	**0.07**	**0.04**
Thiamin, mg/d	1.2	1.5 (0.5)	1.6 (0.6)	1.5 (0.6)	1.5 (0.62)	0.04	0.91
Riboflavin, mg/d	1.2	1.9 (0.6)	2.1 (0.7)	1.9 (0.8)	2.0 (0.8)	0.63	0.77
Niacin, mg/d	14	22.2 (7.6)	22.3 (8.6)	22.2 (7.8)	22.4 (7.4)	0.01	0.95
Vit B6, mg/d	1.6	1.9 (0.9)	2.0 (0.9)	1.9 (0.8)	2.0 (2.0)	0.02	0.72
Folate, μg/d	520	519.4 (218.2)	554.1 (248.7)	557.9 (267.5)	550.3 (287.3)	0.01	0.71
Vit B12, μg/d	2.2	4.8 (3.1)	5.3 (2.8)	5.4 (4.2)	5.2 (2.9)	0.9	0.70
Choline, mg/d **	450 **	**285.1 (160.4)**	**294.0 (150.3)**	**291.5 (208.6)**	**230.4 (138.5)**	**0.11**	**0.005**
**Copper, μg/d**	**800**	**1270 (471)**	**1236 (390)**	**1208 (363)**	**1467 (473)**	**0.14**	**0.0001**
Iron, mg/d	22	14.0 (5.3)	15.1 (5.5)	15.1 (6.0)	15.7 (6.2)	0.003	0.70
**Magnesium, mg/d**	290	280.7 (90.5)	282.4 (93.7)	269.6 (79.4)	326.5 (99.3)	**0.09**	**0.001**
Phosphorus, mg/d	580	1246.6 (391.9)	1262.9 (407.4)	1172.6 (360.1)	1255.3 (353.4)	0.035	0.91
**Selenium, μg/d**	**49**	**108.9 (37.9)**	**101.6 (28.3)**	**100.4 (38.4)**	**88.1 (28.9)**	**0.012**	**0.002**
Zinc, mg/d	9.5	11.2 (4.6)	11.0 (4.0)	10.6 (4.0)	12.2 (4.8)	0.635	0.06
Potassium mg/d	4.7**	2.4 (0.7)	2.4 (0.8)	2.4 (0.8)	2.4 (0.7)	0.003	0.63
**Sodium g/d**	1.5 **	**3.2 (1.0)**	**3.1 (0.9)**	**3.1 (1.0)**	**2.8 (1.1)**	**0.118**	**0.04**

Bold font used to highlight nutrients that statistically differed by randomized group. Abbreviations: TE: total energy; T: time; G × T: group by time, Vitamin A based on RAEs; ^1^ Repeated measures ANOVA adjusted for weeks gestation at randomization, age, ethnicity (Hispanic versus non-Hispanic), parity (multiparity versus primiparity), study entry BMI category (overweight verssu obese), household family income (>50,000/year versus <50,000/year), and baseline value of the variable of interest. However, the mean (SD) values in table are shown unadjusted. ** AI because EARs have not been established; this cutoff is made with less confidence [27,28,29,30,31,32].

**Table 4:** Proportions with micronutrient inadequacy at 35 weeks of gestation by treatment group.

	EAR	Usual Care; *n* = 109 No. (%)		Intervention; *n* = 102 No. (%)					
		Baseline	35 Weeks	Baseline	35 Weeks	Sig ^1^	OR ^1^	95% CI ^1^	
								Lower	Upper
Total Water, L/d; No. (%)	3.0 **	74 (67.9)	72 (66.1)	85 (83.3)	64 (62.8)	0.27	0.70	0.37	1.33
**Calcium, mg/d; No. (%)**	**800**	**43 (39.5)**	**34 (31.2)**	**41 (40.2)**	**18 (17.7)**	**0.007**	**0.37**	**0.18**	**0.76**
CHO, g/d; No. (%)	135	9 (8.3)	9 (8.3)	11 (10.8)	5 (4.9)	0.34	0.55	0.16	1.88
Fiber, g/d; No. (%)	28 **	102 (93.6)	104 (95.4)	98 (96.1)	100 (98.0)	0.32	2.54	0.41	15.91
Added sugars, % TE; No. (%)	<25% of TE	0 (0.00)	5 (4.6)	2 (2.0)	6 (5.9)	0.53	0.52	0.07	4.10
Protein, g/kg/d; No. (%)	0.88	53 (48.6)	71 (65.2)	52 (51.0)	71 (69.6)	0.42	1.30	0.69	2.45
**Vit A, μg_RAE/d; No. (%)**	**550**	**53 (48.6)**	**50 (45.9)**	**44 (43.1)**	**26 (25.5)**	**0.003**	**0.39**	**0.21**	**0.72**
Vit C, mg/d; No. (%)	70	45 (41.3)	56 (51.4)	39 (38.2)	41 (40.2)	0.17	0.66	0.37	1.18
Vit D, μg/d; No. (%)	10	106 (97.3)	103 (94.5)	99 (97.1)	95 (93.1)	0.53	0.68	0.20	2.27
**Vit E, mg/d; No. (%)**	**12**	**98 (89.9)**	**104 (95.4)**	**91 (89.2)**	**79 (77.5)**	**0.001**	**0.17**	**0.06**	**0.48**
**Vit K, μg/d; No. (%)**	90 **	**65 (59.6)**	**70 (64.2)**	**57 (55.9)**	**49 (48.0)**	**0.023**	**0.49**	**0.26**	**0.91**
Thiamin, mg/d; No. (%)	1.2	29 (26.6)	24 (22.0)	38 (37.3)	31 (30.4)	0.08	1.61	0.95	2.73
Riboflavin (mg/d; No. (%)	1.2	10 (9.2)	9 (8.3)	16 (15.7)	10 (9.8)	0.25	1.43	0.77	2.66
Niacin (mg/d; No. (%)	14	12 (11.0)	15 (13.8)	12 (11.8)	11 (10.8)	0.58	1.18	0.66	2.11
Vit B6 (mg/d; No. (%)	1.6	46 (42.2)	44 (40.4)	36 (35.3)	27 (26.5)	0.06	0.56	0.31	1.03
Folate (μg/d; No. (%)	520	63 (57.8)	59 (54.1)	52 (51.0)	55 (53.9)	0.85	1.06	0.59	1.89
Vit B12 (μg/d; No. (%)	2.2	13 (11.9)	8 (7.3)	11 (10.8)	10 (9.8)	0.37	1.62	0.57	4.60
**Choline mg/d**; No. (%)	450 **	**98 (89.9)**	**99 (90.8)**	**91 (89.2)**	**100 (98.0)**	**0.048**	**5.00**	**1.02**	**24.60**
Copper (μg/d; No. (%)	800	13 (11.9)	10 (9.2)	8 (7.8)	7 (6.7)	0.85	1.11	0.37	3.32
Iron (mg/d; No. (%)	22	102 (93.6)	102 (93.6)	90 (88.2)	90 (88.2)	0.55	0.72	0.25	2.08
**Magnesium (mg/d; No. (%))**	290	69 (63.3)	64 (58.7)	70 (68.6)	38 (37.3)	**0.001**	**0.36**	**0.20**	**0.65**
Phosphorus (mg/d; No. (%)	580	1 (0.9)	3 (2.8)	1 (1.0)	3 (2.9)	0.99	1.01	0.18	5.74
Selenium (μg/d; No. (%)	49	2 (1.8)	3 (2.8)	8 (7.8)	9 (8.8)	0.08	3.67	0.88	15.30
Zinc (mg/d; No. (%)	9.5	42 (38.5)	47 (43.1)	44 (43.1)	36 (35.3)	0.25	0.71	0.39	1.27
Potassium mg/d; No. (%)	4.7 **	0 (0)	0 (0)	0 (0)	0 (0)	--	--	--	--
Sodium, g/d; No. (%)	1.5 **	2 (1.8)	2 (1.8)	5 (4.9)	7 (6.9)	0.68	0.91	0.57	1.45

Bold font used to highlight nutrients that statistically differed by randomized group. Abbreviations: ^1^ Logistic regression analysis adjusted for weeks of gestation at randomization, age, ethnicity (Hispanic versus non-Hispanic), parity (multiparity versus primiparity), study entry BMI category (overweight versus obese), household family income (>50,000/year versus <50,000/year), and baseline value of the variable of interest. ** AI because EARs have not been established; this cutoff is made with less confidence [27,28,29,30,31,32].

**Table 5:** Proportions with micronutrient levels above recommended tolerable limits.

	Upper Limit ^1^	Usual Care *n* = 109		Intervention *n* = 102		Sig ^2^	OR ^2^	95% CI	
								Lower	Upper
		Baseline	35 Weeks	Baseline	35 Weeks				
Calcium no., %	2500 (mg/d)	0	0	0	0	--	--	--	--
Vit A no., %	3000 (μg/d)	0	0	0	0	--	--	--	--
Vit C no., %	2000 (mg/d)	0	0	0	0	--	--	--	--
Vit E no., %	1000 (mg/d)	0	0	0	0	--	--	--	--
Niacin no., %	35 (mg/d)	9 (8.2)	5 (4.6)	10 (9.8)	5 (4.9)	0.89	0.91	0.23	3.52
Vit B6 no., %	1000 (mg/d)	0	0	0	0	--	--	--	--
Folate no., %	100 (μg/d)	4 (3.7)	7 (6.4)	8	4 (3.9)	0.10	0.27	0.05	1.30
Choline no., %	3500 mg/d	0	0	0	0	--	--	--	--
Copper no., %	10,000 (μg/d)	0	0	0	0	--	--	--	--
Magnesium no., %	350 (mg/d)	23 (21.1)	24 (22.0)	16 (15.7)	36 (35.3)	0.038	1.97	1.04	3.74
Phosphorus no., %	3500(mg/d)	0	0	0	0	--	--	--	--
Selenium no., %	400 (μg/d)	0	0	0	0	--	--	--	--
Zinc no., %	40 (mg/d)	0	0	0	0	--	--	--	--
Sodium no., %	2.3 g/d	90 (82.6)	85 (78.0)	80 (78.4)	64 (62.7)	0.026	0.47	0.24	0.91

Abbreviations: ^1^ Upper limit (UL) is the highest level of daily nutrient intake that is likely to pose no risk of adverse health effects to almost all pregnant women aged 19–30 years. As intake increases above the UL, the risk for adverse effects increases. ULs are not intended to be a recommended level of intake, rather a level of intake that most individuals can likely tolerate. ^2^ Logistic regression analysis adjusted for weeks of gestation at randomization, age, ethnicity (Hispanic versus non-Hispanic), parity (multiparity versus primiparity), study entry BMI category (overweight versus obese), household family income (>50,000/year versus <50,000/year), baseline value of the variable of interest.

## Supplementary Materials

The following are available online at https://www.mdpi.com/2072-6643/11/5/1071/s1, Table S1: Nutritional Composition of Healthy Beginnings/Comienzos Saludables Meal Replacements.
