# Supplementary material for: Prenatal Intervention with Partial Meal Replacement Improves Micronutrient Intake of Pregnant Women with Obesity
Source: Nutrients. 2019 May 14;11(5):1071. doi: 10.3390/nu11051071 (PMC6567022; doi:10.3390/nu11051071)
Supplement: Supplementary file 1 [file nutrients-11-01071-s001.pdf]

Table S1. Nutritional Composition of Healthy Beginnings/Comienzos Saludables Meal Replacements [1].

Serving size	Organic liquid meal replacement (Orgain, Inc [2]) 330 ml/11 fl oz	Powder meal replacement (Ensure Original Nutrition Powder [3]) 237 ml/8 fl oz	Bar meal replacement (PureFit Bar [4]) 56.7 g
Calories	250	250	230
Total Fat (g)	7	6	7
Sat Fat (g)	1	1	0.5
Cholesterol (mg)	20	<5	0
Sodium (mg)	260	190	150
Potassium (mg)	320	390	140
Total Carb (g)	32	41	24
Fiber (g)	2	1	3
Sugars (g)	12	22	13
Protein (g)	16	9	18
**VITAMINS**			
Vitamin A	25%	25%	0%
Vitamin C	25%	25%	2%
Vitamin D, μg	25%	10%	--
Vitamin E	25%	15%	--
Riboflavin	20%	30%	--
Vitamin B6	15%	25%	--
Vitamin B12	25%	--	--
Pantothenic Acid	25%	40%	--
Thiamine	10%	35%	--
Niacin	20%	15%	--
Folate	25%	25%	--
Biotin	10%	25%	--
**MINERALS**			
Calcium, mg	40%	15%	6%
Iron, mg	2%	10%	20%
Iodine	25%	20%	--
Phosphorus	30%	8%	15%
Magnesium	20%	10%	2%
Copper	25%	15%	--
Zinc	25%	20%	--

Abbreviations: g = grams; mg = milligrams; Sat = saturated; Carb = carbohydrates. [1] Approximately 80% of the study's meal replacements purchases were for the organic meal replacement product (Orgain, Inc); 15% were for the bar meal replacement (PureFit Bar); and, 4% were for the powder meal replacement (Ensure Original Nutrition Powder); the remaining 1% of purchases were for a liquid shake marketed for individuals with diabetes (Glucerna; data not shown). [2] Nutritional information for the organic liquid meal replacement (Orgain) was were accessed on 2/27/19 at: http://orgain.com/products/original-organic-nutritional-shake/. [3] Nutritional information for the powder meal replacement (Ensure Original Nutrition Powder) prepared with water was accessed on 2/27/19 at: https://ensure.com/nutrition-products. [4] Nutritional information for the bar meal replacement (PureFit) was accessed on 2/27/19 at: https://purefit.com/pages/nutritional-information and calculated based on ASA-24 values. Percent Daily Values are based on a 2,000 calorie diet for adults. As nutrient needs tend to be higher during pregnancy, values on the label may be lower for pregnant women.
